# Dataset of daily variation of rain rate distribution at Ota in Southwest Nigeria

**DOI:** 10.1016/j.dib.2019.104154

**Published:** 2019-06-14

**Authors:** Oluwafunmilayo O. Ometan, Temidayo V. Omotosho, Sayo A. Akinwumi, Mustapha O. Adewusi, Adenike O. Boyo

**Affiliations:** a Department of Physics, Covenant University, Ota, Nigeria; b Department of Physics, Lagos State University, Ojo, Lagos State, Nigeria

**Keywords:** Rain rate, Radiowave propagation, Attenuation, Microwave communication

## Abstract

This article presents the data analysis of daily rainfall rate events experienced in Ota, Southwest Nigeria, a tropical station. The meteorological data were measured using a tipping bucket rain gauge mounted at the roof top of the College of Science and Technology, Covenant University, Ota. The data analysed is from April to December 2012. Descriptive statistics were used to show the daily variations in rainfall rate. Hence, the daily variation for rain rate shows variation in the minimum and maximum value within each of the months considered which varies from 0.8 mm/h to 230.4 mm/h. The results from this data will help microwave communication experts have a proper understanding of rainfall rate in this locality. This will assist to ensure a proper and efficient design and planning of radiowave propagation and satellite communication systems in Southwest Nigeria.

**Table:** Specifications table

Subject area	Atmospheric and Communication Environment
More specific subject area	Satellite Communication, Radiowave propagation, Radio Science
Type of data	Table and figures
How data was acquired	Primary Data. The tipping-bucket rain gauge with a resolution of 0.2 mm/tip was used to acquire the data. The rain rate was measured using the tipping bucket to obtain rain rate data at every one-minute integration time. The value of rainfall of 0 mm were filtered out as this represents non-rainy minutes of the day. The remaining rainfall data was sorted and analysed according to the rainfall rate regime.
Data format	Raw and analysed
Experimental factors	Data measurement at Covenant University, Ota, Ogun State, Southwest Nigeria. The automatic weather station comprises of a tipping bucket rain gauge. The rain gauge was installed at a satisfactory height to protect it from high winds and at some distance away from nearby obstacles. This is to prevent any obstacles from blocking the normal path of rainfall into the gauge. This is also to ensure that errors in the data collection is eliminated or possibly reduced which can occur due to the blowing away of rainfall. The bucket is regularly emptied and cleaned to validate that the tipping bucket tips efficiently.
Experimental features	Computational Analysis: Descriptive Statistics
Data source location	Covenant University, Ota, Ogun State, Nigeria (Lat:6.67 ^o^N and Long: 3.23 ^o^E)
Data accessibility	All the data are available in this article

**Table:** 

**Value of the data** • The data will provide a better understanding of the predominant rainfall type in Ota. • The data will be used to calculate an accurate fade margin to mitigate attenuation challenges to communication signals at the location of interest. • The data can be used to extrapolate for other station in Southwest Nigeria where data acquisition is not available, • With the data, microwave engineers will provide reliable communication systems for the location of interest. • The data could be useful for government in understanding of radio propagation within or around the lower atmosphere in the Southwest region of Nigeria.

## 1 Data

**Fig. 1:**
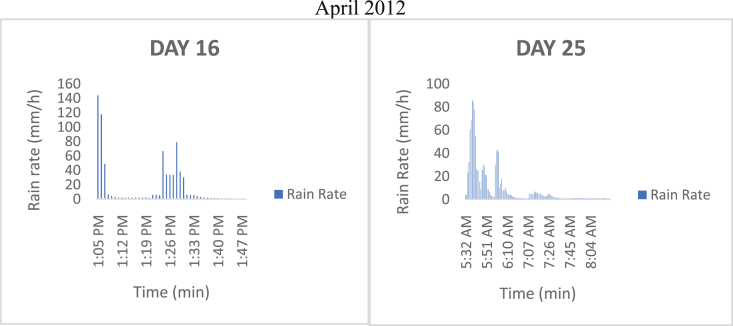
The daily variation in rain rate with time in April 2012.

**Fig. 2:**
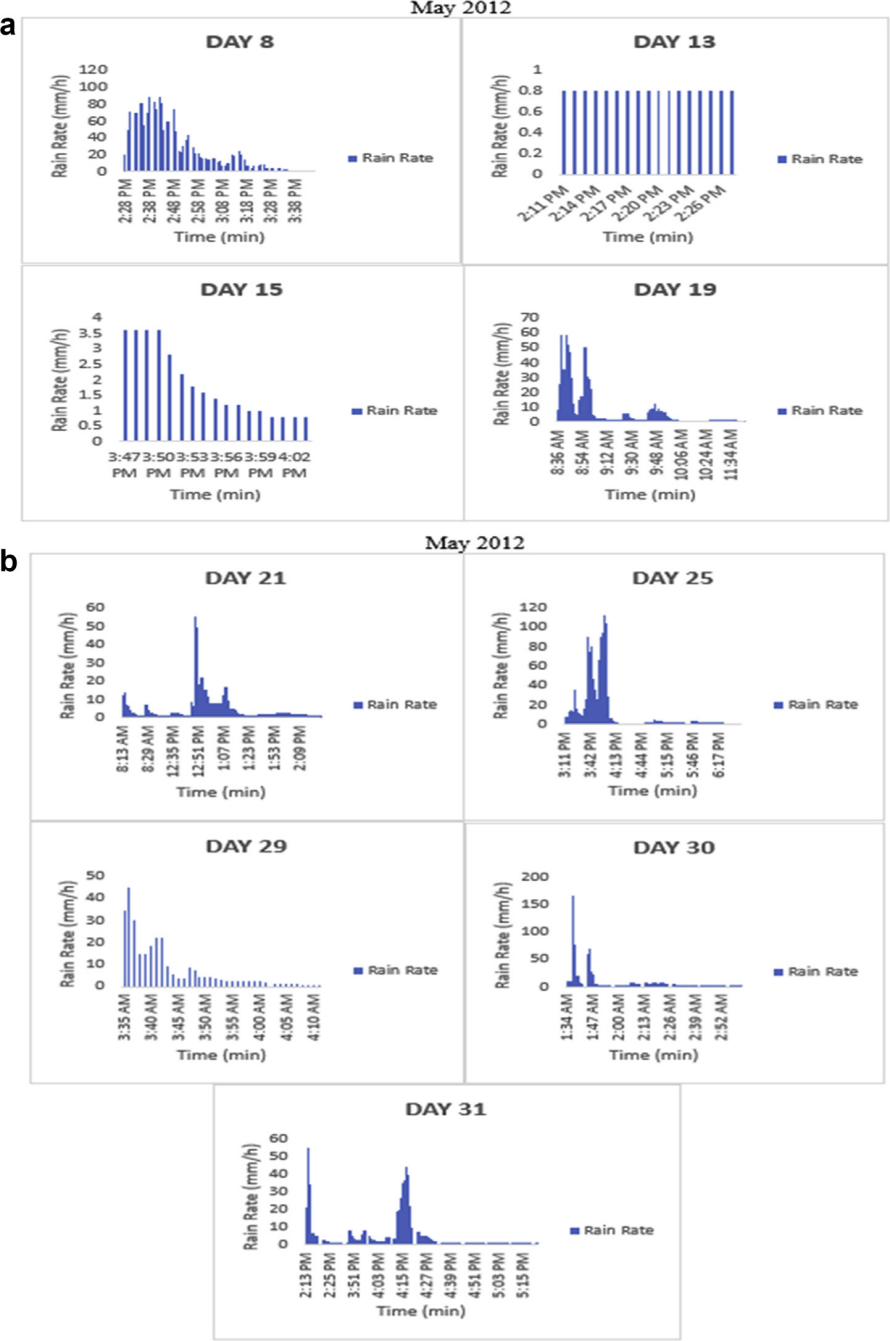
The daily variation in rain rate with time in May 2012.

**Fig. 3:**
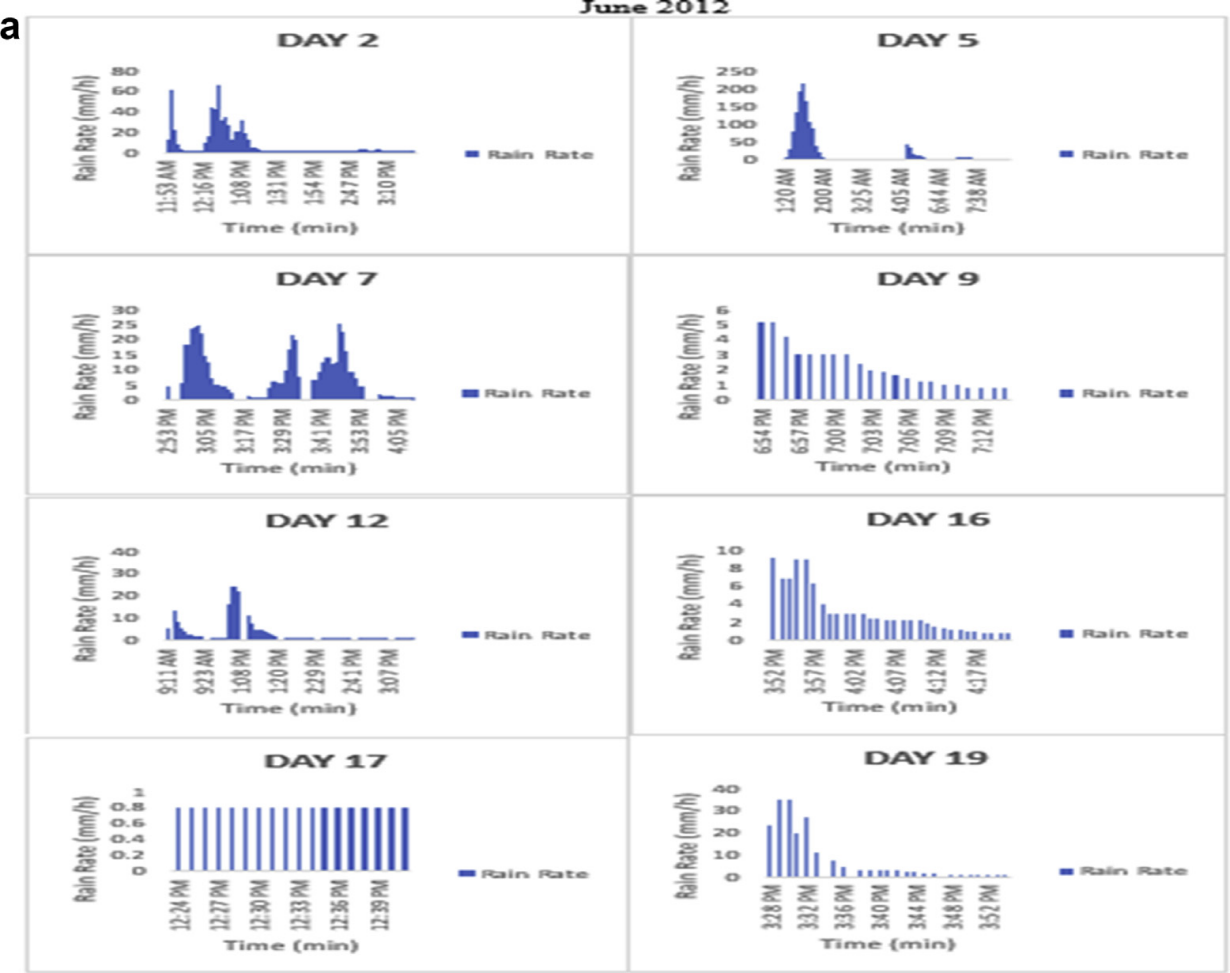

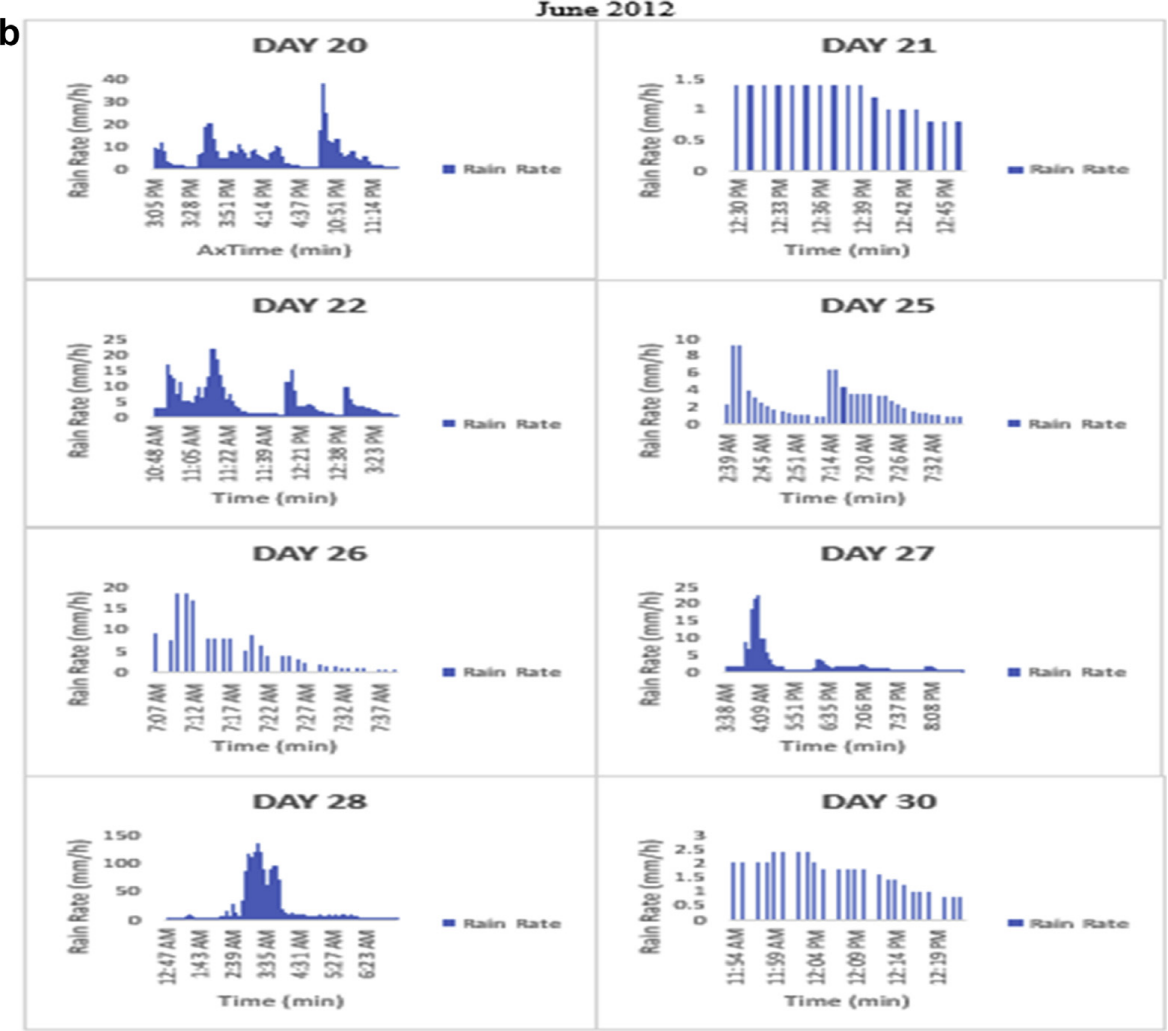
The daily variation in rain rate with time in June 2012.

**Fig. 4:**
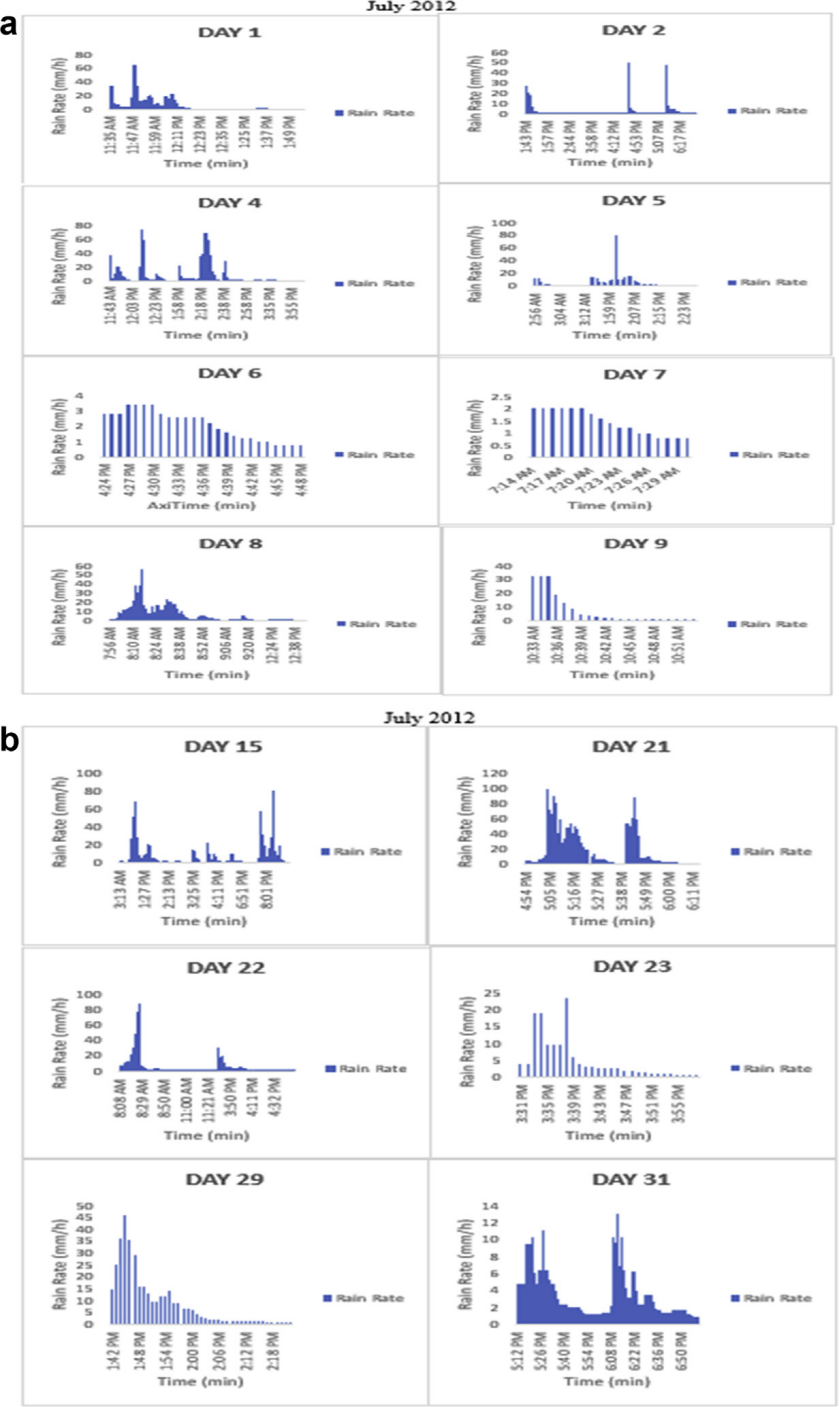
The daily variation in rain rate with time in July 2012.

**Fig. 5:**
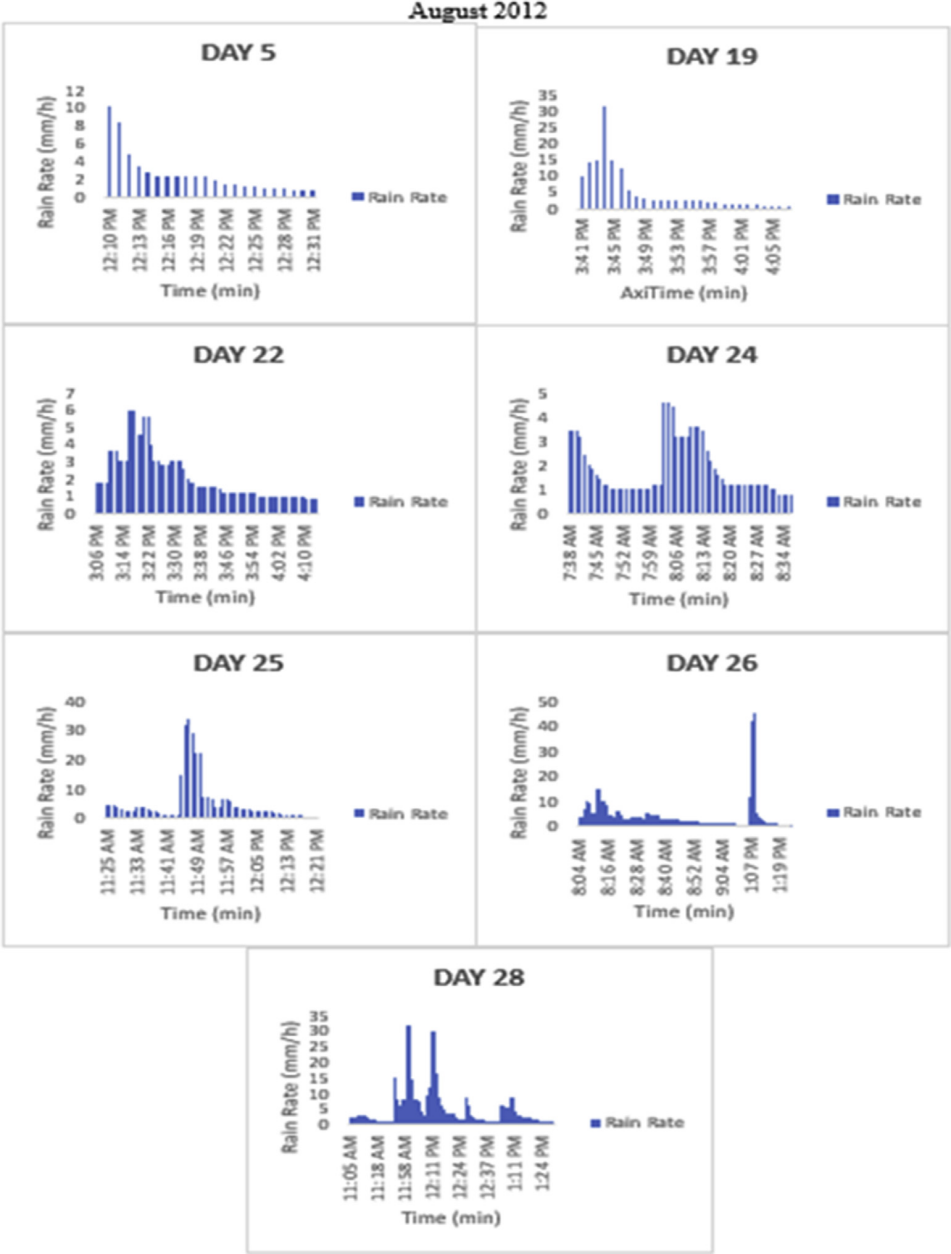
The daily variation in rain rate with time in August 2012.

**Fig. 6:**
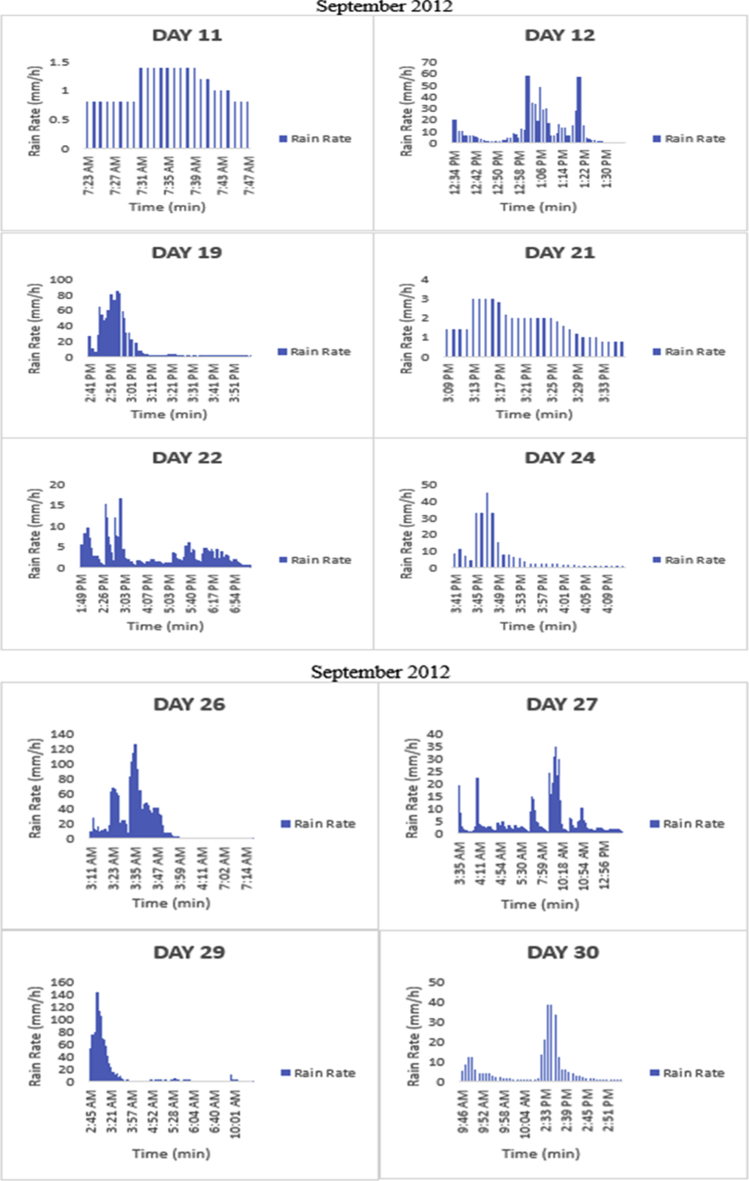
The daily variation in rain rate with time in September 2012.

**Fig. 7:**
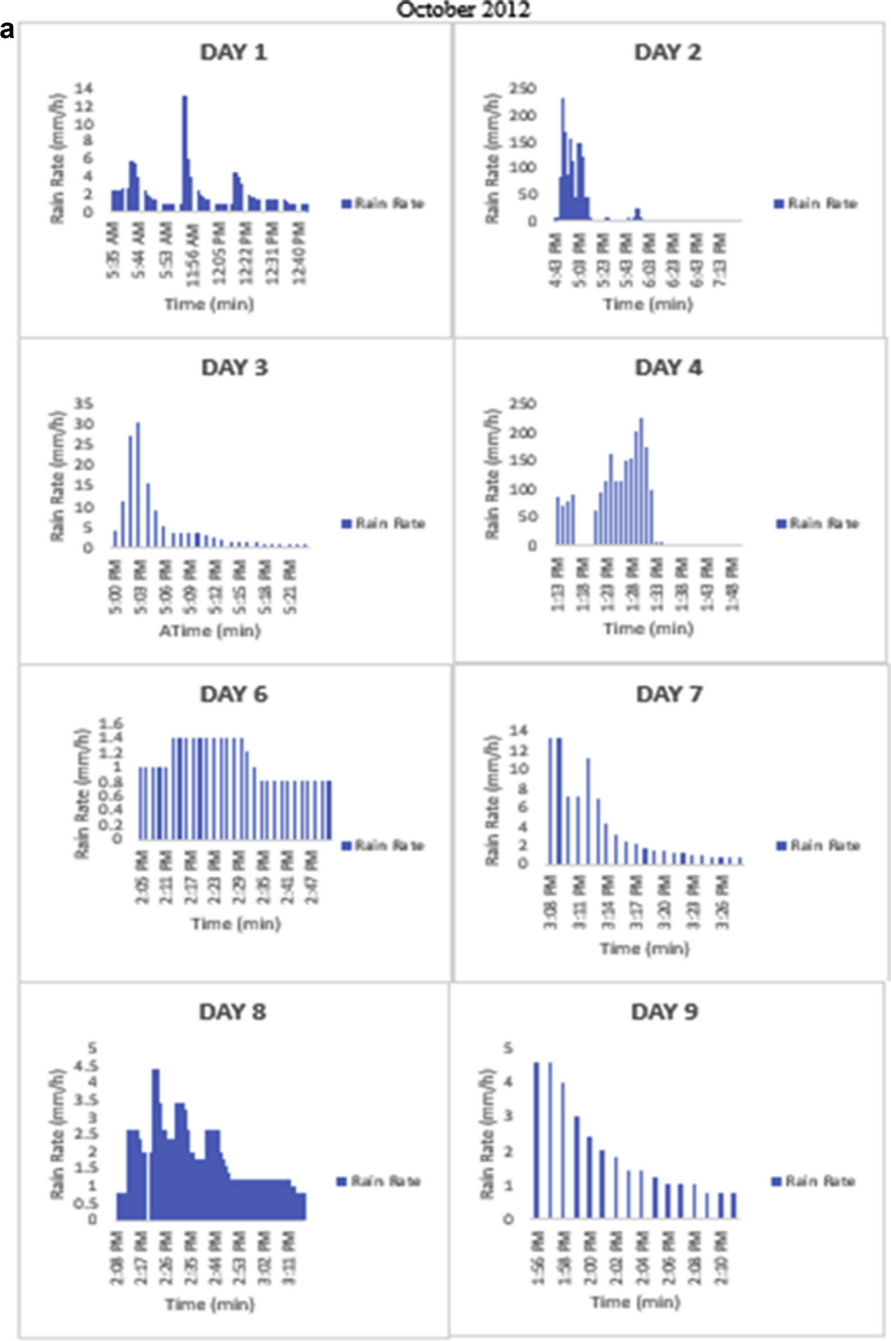

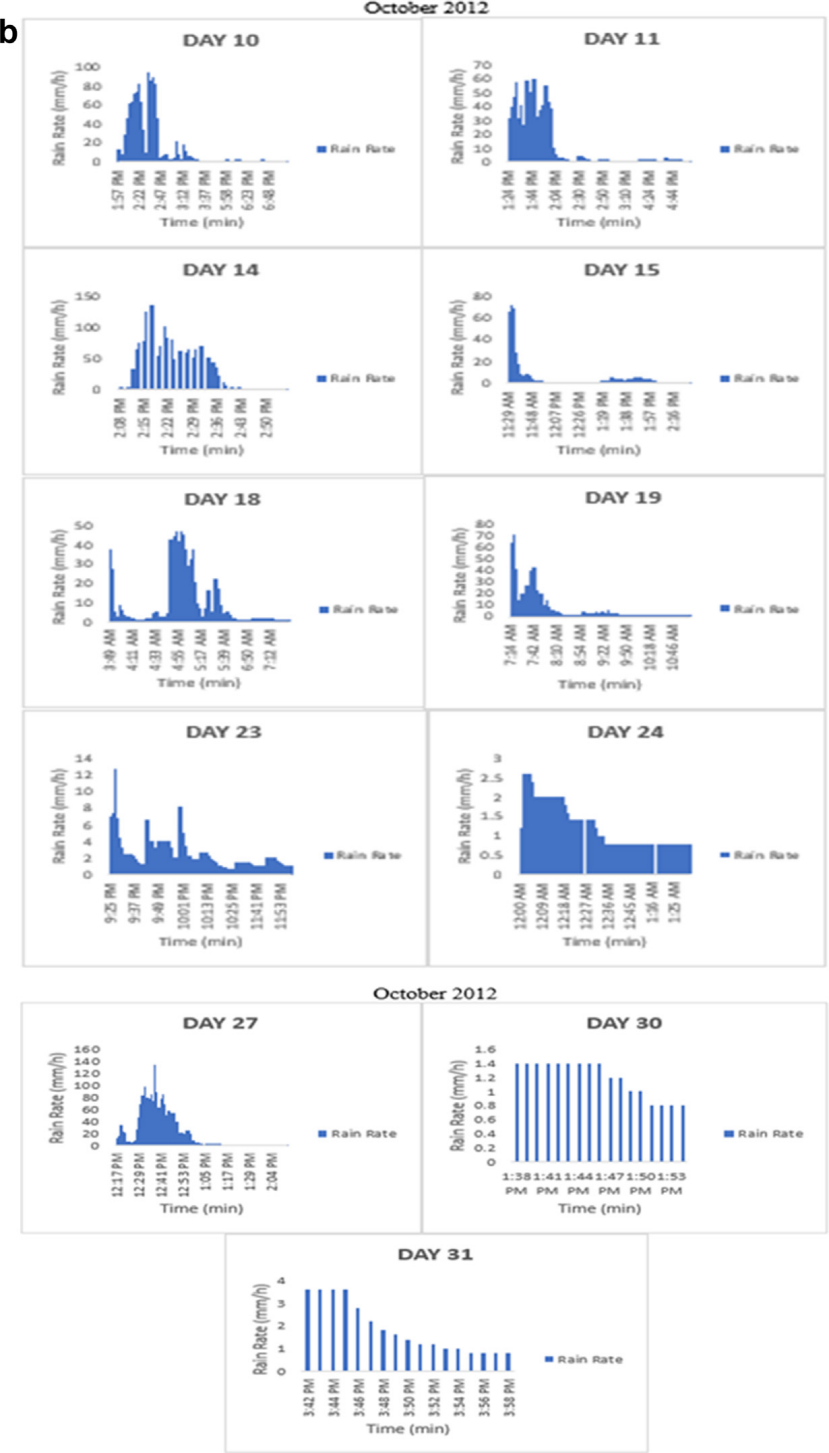
The daily variation in rain rate with time in October 2012.

**Fig. 8:**
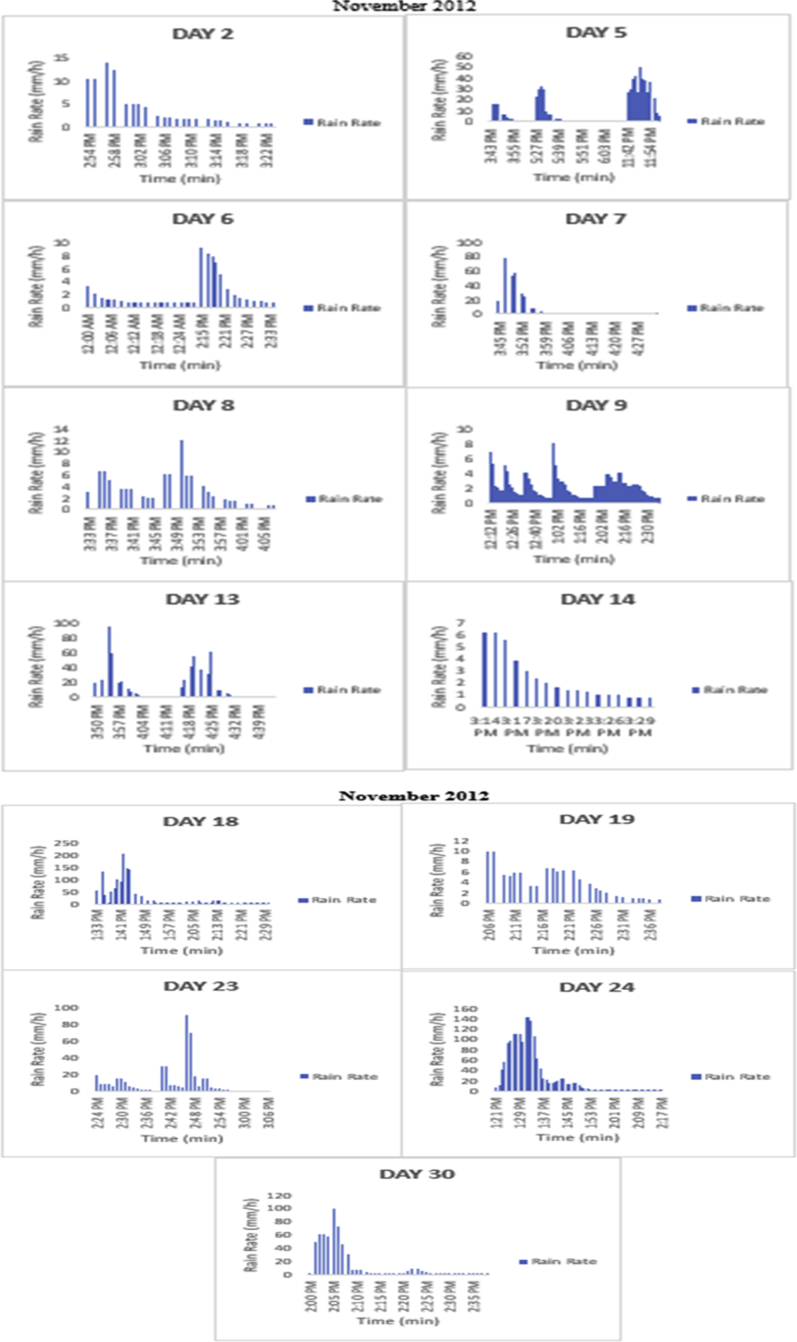
The daily variation in rain rate with time in November 2012.

**Fig. 9:**
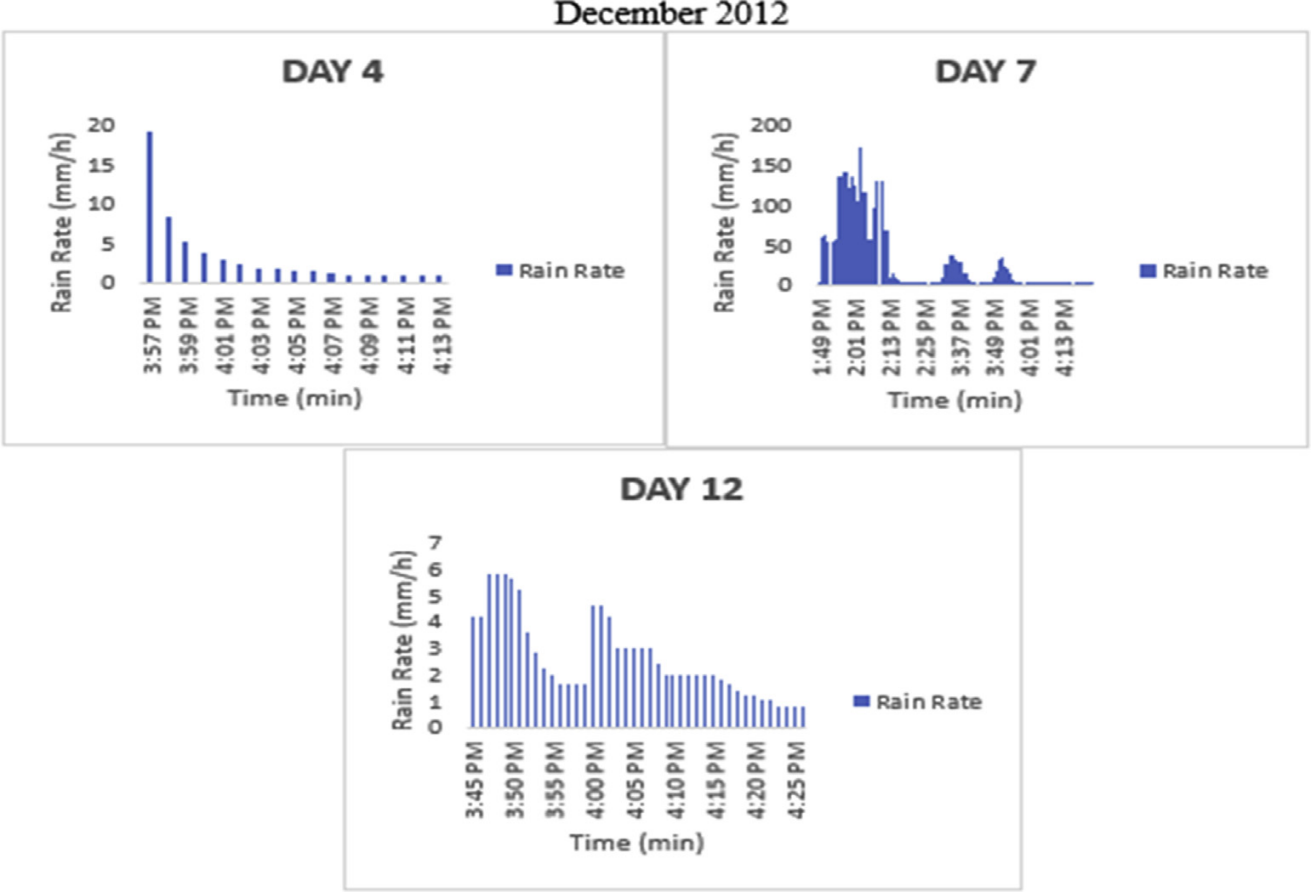
The daily variation in rain rate with time in December 2012.

The meteorological data for this article were measured at Covenant University, Ota, Southwest Nigeria for year 2012. The measurement of the meteorological data was based on one-minute integration time to produce the daily average data and consequently to acquire the monthly data. This helps to understand the daily, monthly, yearly and seasonal variation of Rainfall rate as shown in the tables and figures. The descriptive statistics gives the summaries of the duration of rainfall rate as presented in the Table 1. While, the bar charts show the summaries of the daily distribution of rainfall rate as presented in the Fig. 1, Fig. 2, Fig. 3, Fig. 4, Fig. 5, Fig. 6, Fig. 7, Fig. 8, Fig. 9. Rain rate is the measure of the rate at which rain is falling in a locality. According to [1], precipitation estimates from global maps do not agree with those obtained over Nigeria. Hence, an appropriate distribution of rainfall rate at 1-min integration time is needed for the proper prediction of accurate rain attenuation for the location under consideration. The power law relationship between specific attenuation and rain rate as given in Eq. (1) [2].

(1) $$A=kR^{\alpha }$$

k and α are regression coefficients which depends on drop size distribution, temperature, frequency of propagation and polarization of the radiowave.Where

**Table 1:** Daily duration of rainfall rate in 2012.

Days/Months	Apr	May	Jun	July	Aug	Sep	Oct	Nov	Dec
1				105			67		
2			158	110			160	30	
3							24		
4				172			38		17
5			238	53	22			90	
6				25			48	50	
7			78	17			21	49	95
8		79		120			70	35	
9			21	21			16	107	
10							198		
11						25	157		
12			81			64			42
13		17						55	
14							49	17	
15		17		322			149		
16	44		30						
17			18						
18							175	58	
19		137	27		27	79	220	33	
20			155						
21		126	17	81		28			
22			114	168	69	289			
23				28			91	43	
24					59	32	71	57	
25	133	215	42		79				
26			33		90	89			
27			215			287	94		
28			391		98				
29		37		41		287			
30		89	29			47	17	39	
31		116		110			17

(2) $$\log_{10}k=\sum_{j=i}^{4}a_{j}exp\left[-{\left(\frac{\log_{10}f-b_{j}}{c_{j}}\right)}^{2}\right]+m_{k}\;\log_{10}f+c_{k}$$

and

(3) $$\alpha =\sum_{j=i}^{5}a_{j}exp\left[-{\left(\frac{\log_{10}f-b_{j}}{c_{j}}\right)}^{2}\right]+m_{\alpha }\;\log_{10}f+c_{\alpha }$$

where f is frequency, k is either $k_{H}$ or $k_{v}$ and α is either $\alpha_{H}$ or. $\alpha_{v}$

The data can be used to analyse the percentage of exceedance of the rain rate to show the specific threshold level recorded and exceeded over a period of time.

### 1.1 The summary statistics of the data in 2012

The summary statistics of the data measured in April–December 2012 is presented in Table 1. The data was also presented in a bar chart in Fig. 1, Fig. 2, Fig. 3, Fig. 4, Fig. 5, Fig. 6, Fig. 7, Fig. 8, Fig. 9. The bar chart is a representation of the descriptive statistics which reveals the variation of rainfall rate for some of the months in the year.

## 2 Experimental design, materials, and methods

The measurement was conducted at Covenant University, Ota, (6.67◦N, 3.23◦E), Southwest Nigeria. The in-situ precipitation measurement started in April 2012 and is ongoing. The data used for this analysis is of a period of 9 months. The data logger (Davis instrument Weather Link) employed for capturing and harvesting the data is connected to a personal computer (PC) for data harvesting. The rainfall data was analysed by sorting and classifying the rainfall rate into four different categories, namely: the drizzle rain type (below 5 mm/h); the widespread rain type (between 5 mm/h and 10 mm/h); the shower rain type (between 10 and 40 mm/h) and the thunderstorm rain type (above 40 mm/h). Although, both rainfall and non-rainfall measurements are ongoing by some researchers in Nigeria [3], [4], [5], [6], [7], [8], [9], [10], [11], [12], [13]. Some descriptive statistics data measurement was also used by [14], [15]. Moreover, this research work is one of the few in Southwest Nigeria. The data will serve as a great tool for the analysis and prediction of rain attenuation and the extent of its impairment on communication signals in the tropics.
